# Epigenetic regulation of fetal bone development and placental transfer of nutrients: progress for osteoporosis

**DOI:** 10.2478/v10102-011-0026-6

**Published:** 2011-12

**Authors:** Georgeta Bocheva, Nadka Boyadjieva

**Affiliations:** 1 Department of Pharmacology and Toxicology, Medical Faculty, Medical University, Sofia, Bulgaria; 2 Endocrine Research Facility, Department of Animal Sciences, Rutgers University, New Brunswick, New Jersey, 08901, USA

**Keywords:** osteoporosis, osteogenesis, epigenomics, vitamin D

## Abstract

Osteoporosis is a common age-related disorder and causes acute and long-term disability and economic cost. Many factors influence the accumulation of bone minerals, including heredity, diet, physical activity, gender, endocrine functions, and risk factors such as alcohol, drug abuse, some pharmacological drugs or cigarette smoking. The pathology of bone development during intrauterine life is a factor for osteoporosis. Moreover, the placental transfer of nutrients plays an important role in the building of bones of fetuses. The importance of maternal calcium intake and vitamin D status are highlighted in this review. Various environmental factors including nutrition state or maternal stress may affect the epigenetic state of a number of genes during fetal development of bones. Histone modifications as histone hypomethylation, histone hypermethylation, hypoacetylation, etc. are involved in chromatin remodeling, known to contribute to the epigenetic landscape of chromosomes, and play roles in both fetal bone development and osteoporosis. This review will give an overview of epigenetic modulation of bone development and placental transfer of nutrients. In addition, the data from animal and human studies support the role of epigenetic modulation of calcium and vitamin D in the pathogenesis of osteoporosis. We review the evidence suggesting that various genes are involved in regulation of osteoclast formation and differentiation by osteoblasts and stem cells. Epigenetic changes in growth factors as well as cytokines play a rol in fetal bone development. On balance, the data suggest that there is a link between epigenetic changes in placental transfer of nutrients, including calcium and vitamin D, abnormal intrauterine bone development and pathogenesis of osteoporosis.

## Introduction

Osteoporosis is a common age-related disorder. It is characterized by an excessively fragile skeleton and susceptibility to fractures (Harvey *et al.*, 2008). Osteoporosis manifests clinically by fractures of the hip, vertebrae, distal forearm, etc. and the major cause of these fractures is low bone mass. It is well established that the skeletal size and density increase from early embryogenesis through intrauterine, infant, childhood and adult life. Various gene-mapping approaches have been applied to identify specific genes during all periods of bone development. Moreover, genes were detected for osteoporosis, involving largely low bone mineral density (BMD), which is one of the strongest risk factors for osteoporotic fracture. Gene studies focused on BMD and documented the phenotype genetic correlation between BMD and osteoporotic fracture (Bass *et al.*, 1998). Twin and family studies have shown that genetic factors play an important role in osteoporosis. Progress was made in: 1) heritability, osteoporosis and phenotype studies; 2) approaches for gene mapping and identification of gene mutations for osteoporosis; 3) candidate gene association studies; 4) nutrients and osteoporosis; and 5) pharmacological drugs and pharmacogenomic studies for osteoporosis (Long *et al.*, 2005).

Epigenetic regulation of bones and osteoporosis is not well established. Moreover, the effect of nutrition status of the mother has been discussed for many years and several studies considered it as an environmental epigenetic factor that may play an important role during fetal development. Nutrition, especially calcium intake influences the building of bones and prophylaxis of osteoporosis. Mother-offspring studies have demonstrated the role of some specific factors in early life, such as maternal body build, lifestyle and 25(OH)-vitamin D status of the mother, which might be important for bones of the fetus/neonate. In addition, the placenta plays a role in the nutrition status of the developing fetus. It is involved in the transport of nutrients and waste products between mother and fetus, being a source of many peptides and hormones that affect fetal development (Burton *et al.*, 2001). The aim of the present review is to analyze published data in the field of epigenetic regulation of placental transfer of nutrients which may support better prophylaxis and treatment of osteoporosis. This review also summarizes the current knowledge on epigenetics of growth factors, cytokines and hormones in fetal bone development and their impact on osteoporosis.

## Basic genetic characteristics of bone development and osteoporosis

Regulatory regions of the human genome can be modified through epigenetic processes during prenatal life. Genetic susceptibility to osteoporosis may be both context dependent and developmentally regulated, and epigenetic mechanisms are the likely link between gene and environment. Epigenetic changes may make individuals more likely to suffer from chronic diseases, as osteoporosis, when they reach adulthood. The modification of chromatin and DNA contributes to a larger well-documented process known as “programing”.

Osteoporosis is one of various well documented “programing” chronic diseases (Tang *et al.*, 2007). Chromosome 11q12-13 has been investigated for its linkage to normal BMD variation (Koller *et al.*, 2000; 2001). Reneland *et al.*(2005) investigated more than 25,000 single-nucleotide polymorphisms (SNPs) located within 16,000 genes and established that variants in the gene encoding the phosphodiesterase 4D account for some of the genetic contribution to BMD variation in humans. The cause for osteoporosis pseudoglioma is a mutation in *LRP5* (low-density lipoprotein receptor-related protein), which is a member of Wnt family genes. Many of the known genes implicated by the 29 loci identified through GWAS participate in the Wnt/β-catenin signaling pathway. It is one of the signaling pathways through which members of the Wnt family can signal (*e.g. GPR177*, *CTNNB1*, *LRP5*, and *SFRP4)* and regulate bone development and bone loss. Xiong *et al.*(2009) reported the results from genome-wide association and follow-up replication studies which identified additional genes *ADAMTS18* and *TGFBR3* as bone mass candidate genes in different ethnic groups (Xiong *et al.*, 2009). In addition, the *RANKL*/*RANK*/*OPG* pathway has also emerged from candidate gene and GWAS of BMD (*e.g. TNFSF11* and *TNFRSF11B*). It is known that this pathway is involved in the regulation of osteoclast formation and differentiation by osteoblasts and stromal stem cells. The signaling system that involves receptor activation of nuclear factor-κB (RANK) by its ligand (RANKL), which is regulated by the soluble decoy receptor osteoprotegerin (OPG), plays a role in the *RANKL*/*RANK*/*OPG* pathway of gene control of fetal development of bones and osteoporosis.

**Figure 1:**
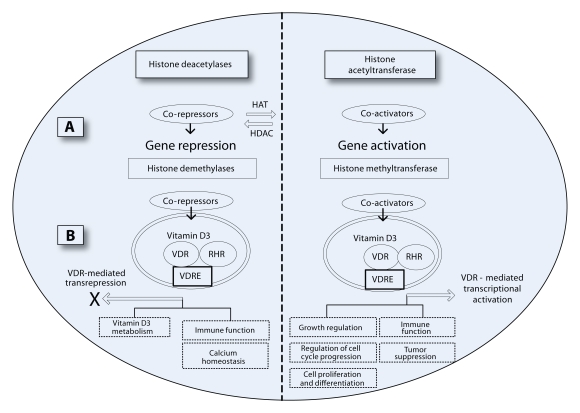
Epigenetic mechanism via modifications of histone enzymes (deacetylases, demethylases, acetyltransferase or methyltransferase) plays a role in vitamin D3 (1α,25-dihydroxyvitamin D3)-mediated regulation of cellular and physiological functions. Histone acetylation correlates with active gene transcription, and deacetylation associated with gene repression. Histone demethylases are involved in vitamin D3 receptor (VDR)-mediated transrepression. Gene repression of vitamin D3 and VDR affects calcium homeostasis, immune function as well as metabolism of vitamin D3. Histone methyltransferase is involved in vitamin D3-mediated cell growth, cell cycle progression, cell proliferation and differentiation, tumor suppression and immune function. VDR-mediated transcriptional activation plays a role in all the above vitamin D3-mediated cellular functions. Histone modifications as histone hypomethylation, histone hypermethylation, histone hypoacetylation, etc. may affect intrauterine growth regulation of bones.

The maintenance of calcium homeostasis by parathyroid hormone (PTH) is well established. Various animal and human studies have shown that the parathyroid gland secretes PTH in response to low serum calcium concentrations, which acts to normalize calcium levels through several mechanisms, including promotion of bone absorption (Nissenson *et al.*, 2009). Some evidence has suggested that genes connected with PTH are strong candidate for genetic regulation of bone development and bone loss. In addition, several studies reported polymorphisms in genes in the PTH pathway. PTH, PTH-like hormone (*PTHLH*), and PTH1 receptor (*PTHR1*) are associated with either fracture risk or BMD. Their involvement in fetal bone development is not yet clear (Scillitani et al, 2006; Vilariño-Güell *et al.*, 2007; Gupta *et al.*, 2008). Additional candidate for fetal bone development and osteoporosis is the gene *COL1A1 Sp1* for type 1 collagen, which is a major protein of bone. The polymorphism of *COL1A1 Sp1* has been associated with osteoporotic fractures and BMD. Moreover, the studies suggest that *COL1A1 Sp1* plays a role in fetal bone development (Ralston, 2010). The vitamin D receptor (*VDR*) gene has been studied for its role in bone metabolism. The studies of Ensrud *et al.* (2009) evaluated the relation of *VDR* polymorphisms to bone loss. They found that low 25-hydroxyvitamin D levels have were associated with a higher rate of decline in total hip BMD in men. Several studies have reported associations between polymorphisms in *VDR* and cross-sectional measures of BMD (Cooper *et al.*, 1996). On balance, the data suggest that the gene for VDR receptor plays a role in fetal bone development. Figure 1 shows the role of VDR gene in regulation of various functions, as cell growth regulation, cell proliferation and differentiation, immune functions, calcium homeostasis, etc., which are important for bones (Sundar and Rahman, 2011). In addition, various transcription factors that play roles in bone formation have been identified. For example, Sox9, Runx2, and Osterix are active in osteoblasts (Baek and Kim, 2011). In addition, the osteogenic genes such as ALP, type I collagen, osteocalcin, and bone sialoprotein (Bsp) are essential for osteoblast differentiation and bone formation (Matsubara *et al.*, 2008).

## Epigenetics – link between genes and the environment in regulation of bone development and osteoporosis

Epigenetic changes are modifications to the DNA, which can be caused by various factors including environment, and which do not change the underlying sequence. Histone modifications, as histone hypomethylation, histone hypermethylation and hypoacetylation, etc., are involved in chromatin remodeling known to contribute to the epigenetic landscape of chromosomes. Epigenetic modifications can be passed down from the mother or father and can influence even the grandchild generation. They have the capacity to control whether a gene is turned on or off or how much message is processed. These changes to gene expression can lead to dramatic differences in individual characteristics. Disruption of the epigenome is a fundamental mechanism in fetal development diseases. Epigenetic modifications, leading to dysregulation of critical genes controlling cell proliferation, differentiation, and death of cells in fetal bones are not well established. However, epigenetics is a rapidly expanding field and various studies demonstrated the role of epigenetic modifications in bone development and osteoporosis. It has now been shown that the birth weight and childhood growth of 7,000 men and women born in Helsinki University Central Hospital during 1924–1933 were linked to hospital discharge records for hip fracture (Coopere *et al.*, 2001). The results from the Helsinki Cohort Study suggest that the growth *in utero* and early life of the offspring is associated with the actual risk of fracture. Twin studies also provided various opportunities to study the effect of the environment upon development, while controlling genetic variation (Javaid *et al.*, 2004). In a study of 445 monozygotic and 966 dizygotic twins from the UK, at a mean age of 47 years, birth weight was found to positively predict BMC and BMD (Antoniades *et al.*, 2003). The results suggest that there are epigenetic modifications in the regulation of bone development.

Epigenetics refers to stable and heritable changes in gene expression that do not involve changes in DNA sequence. It is well established that histones stabilize DNA. Many environmental factors interact with genes through epigenetic mechanisms in human diseases, including osteoporosis (Hunter, 2005). Gene-environment interactions are involved in fetal bone development and the changes may cause osteoporosis later in life. A specific genotype may result in a phenotype only in certain environments. For example, the interactions between the genome and fetal environment might establish basal levels of circulating growth hormone and contribute to accelerated bone loss. Many of these interactions occur primarily during early life (in fetuses and neonates). Several environmental factors have been shown to directly influence methylation patterns and expression levels of some genes. Moreover, in some cases, epigenetic alterations are transmissible beyond a single generation (Jiang *et al.*, 2004). Prenatal glucocorticosteroids and neonatal behavioral manipulation (especially stress) promoted changes in histone acetylation and DNA methylation in a transcriptional factor binding site of glucocorticoid receptor (Weaver *et al.*, 2004). The studies cited above on 3,639 men and 3,447 women born in Helsinki University Central Hospital supported the motion of epigenetic regulation of bone development. The results demonstrated that after adjustment for age and gender, there were two major determinants of hip fracture risk: tall maternal height and low rate of childhood growth. The data also showed that the hip fracture risk was elevated among babies born short. Fracture subjects were shorter at birth but of average height by the age of 7 years. These data suggest that the epigenetic mechanisms that respond to *in utero* or early life exposures play a critical role in growth/development of bones. The studies on animals also support the interactions between environmental factors and epigenetic mechanisms in bone development and osteoporosis. It was shown that alterations to the diet of pregnant animals can produce lasting changes in the offspring's physiology and metabolism (Bateson, 2001). During mammalian development, signals about the nutritional status transferred from the mother to her embryo or fetus either through the placenta or through lactation can affect the epigenetic reprograming in mammals (Morgan *et al.*, 2005). This transfer of signals from mother to fetus may act to limit fetal growth, in preference to any genetic potential, and has been named ‘maternal constraint’ (Gluckman *et al.*, 2008). Poor diet or limited nutrients for the fetus may cause various changes, including epigenetic modifications of histones/genes involved in bone development. In addition, stressful stimuli and cortisol are major factors for epigenetic changes of fetal development. It was documented that the profiles of endogenous circulating cortisol and bone mineral density in healthy elderly men are dependent on fetal growth (Dennison *et al.*, 1999). Moreover, a statistically significant positive association was found between characteristics of cortisol concentration and femoral neck bone loss rate in 22 men, aged 61–72 years, undergoing replicate bone density measurements over a 4-year period. Their data also suggest that the endogenous cortisol profile of healthy elderly men is a determinant of their rate of fetal bone loss.

On balance, the results from human and animal studies suggest that both nutrients and environmental factors as stress, smoking, drug abuse, radiation, etc. may play a role in epigenetic changes of intrauterine bone development.

## Placental transfer of nutrients: epigenetic regulations and their role in fetal bone development and osteoporosis

DNA methylation, histone modifications and non-coding RNAs, affect gene expression in the placenta. This expression, including the important parent-of-origin-dependent gene expression resulting from genomic imprinting, plays a role in both fetal and placental development (Nelissen *et al.*, 2011). The study showed that the epigenetic of the placenta is a major factor of placental supply of nutrients to the fetus. Calcium and vitamin D are key nutrients likely to influence fetal bone development. The human fetus requires a total of 30 g of calcium for bone development, most of which is acquired during the third trimester via active transport across the placenta. Fetal calcium needs are primarily met by increased maternal intestinal calcium absorption during pregnancy. The data indicated that very low maternal calcium intake may be a risk for lower bone mass in fetuses as well as in neonates. The importance of maternal vitamin D status has also been studied (Namgung and Tsang, 2003). It is well established that 1,25(OH)-vitamin D (the active form) mediates vitamin D effects first by binding to the vitamin D receptors (VDR), then by binding to the retinoic acid receptor (RXR), forming a heterodimer (Kimball *et al.*, 2008). This heterodimer then acts upon vitamin D response elements in target genes, initiating gene transcription by either up-regulating or down-regulating gene products. The expression of a placental calcium transporter (PMCA3) gene predicts neonatal whole-body BMC (Martin *et al.*, 2007). The effects of maternal nutrition target the promoter region of specific genes. The VDR, the collagen type I alpha 1 gene (COLIA1) and estrogen receptor gene (ER) alpha have been most widely investigated and found to play a role in regulating BMD as well as targets for epigenetic regulation of placental transfer of nutrients (Williams and Spector, 2006). Epigenetic changes may associate with changes in DNA methylation of genes within or located near to vitamin D response elements. Modified expression of the genes encoding placental calcium transporters by epigenetic regulation may indicates data that maternal vitamin D status influences bone mineral homeostasis in the neonate. WNT2 promoter methylation in the human placenta was found to be associated with low birth weight percentile in the neonate (Ferreira *et al.*, 2011).

The DNA methylation differences identified in the promoter of the WNT2 gene were studied in an extended cohort of 170 samples. The results demonstrated that the WNT2 gene plays an important role in mouse placental development and confirmed its high expression in the human placenta. High WNT2 promoter methylation (WNT2PrMe) was found only in placental tissue and not in the cord blood of the fetus. The results also showed that WNT2 expression can be epigenetically down-regulated in the placenta by DNA methylation of its promoter and that high WNT2PrMe is an epigenetic variant associated with reduced fetal growth potential. Dysregulation of the genes led to the hypothesis that various epigenetic changes in the placenta responsible for nutrients transfer to the fetus played roles in abnormal fetal bone development and contributed to osteoporosis in the adults. Moreover, the results support the view that low birth weight as well as low BMD may result from maternal, fetal, placental and environmental factors.

In addition, various studies suggest that the epigenetic modulation of the HPA axis represents a second mechanism by which poor maternal nutrition as well as environment may cause epigenetic changes in bones of fetuses and neonates. Protein restriction during mid and late pregnancy was found to be associated with reduced methylation of key CpG-rich islands in the promoter region of the gene for the glucocorticoid recpetor (GR) (Weaver *et al.*, 2004). Further there was increased expression of the GR and DNA methylation, which is maintained through mitosis by DNA methyltransferase-1 (Dnmt1) activity. Moreover, the phenotype of an embryo can be modified by manipulation of DNA methyltransferase-1 (Dnmt1) expression by DNA methylation (Biniszkiewicz *et al.*, 2002; Bird, 2001). In addition, several studies have confirmed that independent predictors of greater neonatal whole-body bone area and BMC include genes and their role in regulation of greater maternal birth weight, height and fat stores. Maternal activities such as smoking or drug abuse, dietary choices including calcium and vitamin D intake, and endocrine functions are factors for offspring bone development and may play roles in epigenetic mechanisms of placental transport of nutrients (Dennison *et al.*, 1999, 2004; Javaid *et al.*, 2004, 2005). Thus, for example, data that maternal smoking is statistically significantly associated with lower neonatal bone mass suggest a possible role of epigenetic changes.

## Epigenetic pathways in bone development in animal and human models

Rat dietary protein restriction leads to altered Dnmt1 expression. Dnmt1 expression in human umbilical cord predicts GR1-C_total_ (human glucocorticoid receptor 1-C_total_ promoter) methylation. It was established that GR1-C total methylation status in human umbilical cord predicts GR expression. Induction of altered epigenetic regulation of the hepatic glucocorticoid receptor in the offspring of rats fed a protein-restricted diet during pregnancy suggests that reduced DNA methyltransferase-1 expression is involved in impaired DNA methylation and changes in histone modifications (Lillycrop *et al.*, 2007). Cooney *et al.* (2002) found that maternal methyl supplements in mice affected epigenetic variation and DNA methylation of the offspring. In the agouti mouse mutant, maternal dietary folate supplementation at conception altered the expression of the imprinted agouti gene by altering the capacity for methylation (Cooney *et al.*, 2002). The relationship between poor nutrition *in* *utero*, weight in infancy and adult BMD has been replicated in population studies in the United States, Australia and Scandinavia (Cooper *et al.*, 2006). These results suggest that genetic or epigenetic influences on bone development may be modified by poor nutrition *in utero.* Studies in 198 children aged 9 years demonstrated that lower preconception maternal weight, reduced maternal fat stores during late pregnancy, a history of maternal smoking during pregnancy and lower maternal social class were all associated with reduced whole-body BMC of the child at the age of 9 years (Javaid *et al.*, 2006). Moreover, a lower ionized calcium concentration in umbilical venous serum may predict reduced fetus and neonate bone mass. Around 31% of the mothers had insufficient and 18% had deficient circulating concentrations of 25(OH)-vitamin D during late pregnancy. Lower concentrations of serum 25(OH)-vitamin D in mothers during late pregnancy were associated with reduced whole-body and lumbar spine BMC in children at the age of 9 years.

Growth factors (GF) play important roles in bone development (Winsloe *et al.*, 2009). IGF-2 is known to be a key factor in human growth and development and it was found to be associated with epigenetic changes Interestingly, IGF-2 gene was hypomethylated, whereas interleukin-10, leptin, ATP-binding cassette A1 and maternally expressed 3 (meg 3) genes were hypermethylated. This study further supports the importance of investigating how early epigenetic modification of gene expression may influence long-term health and disease.

IGF2 and H19, belonging to the same cluster of imprinted genes and regulated by ICR1, DMR2 and H19 promoter elements, play a major role in fetal/placental growth. The epigenetic modulation of IGF2/H19 during human development was marked in 60 normal and 66 idiopathic IUGR (Intrauterine Growth Restriction) pregnancies. Embryonic (cord blood) and extraembryonic (placenta and umbilical cord) tissues were studied. Normal ICR1 methylation levels (approximately 50%) and H19 promoter/DMR2 hypomethylation were found in extra-embryonic tissues. In contrast, in embryonic samples the three loci displayed normal methylation values comparable to those in postnatal blood. Asymmetric allelic expression of H19 and IGF2 was reported as a common feature in pre- and post-natal tissues, independent of H19 promoter and DMR2 methylation levels. The study suggests that the gradient of global methylation, increasing from extra-embryonic to embryonic and adult tissues, might play an important role in fetal/bone development. In addition, hypomethylation of H19 promoter and DMR2 were not found to influence the expression pattern of IGF2 and H19 (Tabano *et al.*, 2011).

The role of cytokines in epigenetic regulation of bones is still not well established. There are data indicating that IL-6 genetic variation is prominently associated with hip BMD in late postmenopausal women without estrogen replacement therapy and/or with inadequate calcium intake (Ferrari *et al.*, 2004). Growth factors interact with various cytokines in regulation of fetal development and further studies will determine whether the epigenetic modulations play roles in GF/cytokine relations.

## Conclusion

Osteoporosis is a complex disease determined by genetic and environmental factors, as well as possible interactions among these factors. Epigenetic regulation of placental transfer of nutrients plays a role in both bone development and osteoporosis. Hypermethylation or hypomethylation, hypoacetylation or hyperacetylation of genes were found to regulate calcium, vitamin D, cytokines, growth factors, etc. in bone development.
